# Evaluation of the Decrease in DPOAE Levels After VEMP Testing in Clinical Patients Referred to the Vertigo Outpatient Clinic

**DOI:** 10.3390/jcm14082766

**Published:** 2025-04-17

**Authors:** Shinnosuke Asakura, Teru Kamogashira, Hideaki Funayama, Toshitaka Kataoka, Shizuka Shoji, Megumi Koizumi, Shinichi Ishimoto, Tatsuya Yamasoba

**Affiliations:** 1 Department of Clinical Examination, JR Tokyo General Hospital, Tokyo 151-8528, Japan; 2 Department of Otolaryngology, JR Tokyo General Hospital, Tokyo 151-8528, Japan; 3 Department of Otolaryngology and Head and Neck Surgery, Faculty of Medicine, University of Tokyo, Tokyo 113-8655, Japan; 4 Department of Otolaryngology, Tokyo Teishin Hospital, Tokyo 102-8798, Japan

**Keywords:** DPOAE, VEMP, sensorineural hearing loss, SNHL, noise-induced hearing loss, NIHL

## Abstract

**Background/Objectives**: The objective of this study is to determine whether the strong acoustic stimuli used in vestibular evoked myogenic potential (VEMP) testing contribute to distortion product otoacoustic emission (DPOAE) level reduction due to noise-induced hearing loss. **Methods**: The DPOAE levels were measured routinely to evaluate vestibular balance disorders with sensorineural hearing loss and to monitor changes in cochlear function before and after VEMP. The changes in DPOAE levels after VEMP testing in 174 patients (80 males and 94 females; median age, 53 years [interquartile range, 39–67 years; range, 15–85 years]) who were examined in the vertigo outpatient clinic between June 2021 and December 2024 were retrospectively analyzed. **Results**: The DPOAE levels decreased significantly after VEMP testing at 1.4 kHz, 2 kHz, 2.8 kHz, sum all 1/2 octave, and average 1/2 octave (1–6 kHz). The decrease in DPOAE levels at 6 kHz exhibited a significant negative linear correlation with age (the coefficient of determination: 0.0189, *p* = 0.01), but not sex or side. **Conclusions**: The strong sound stimulation used in VEMP testing can decrease DPOAE levels. The frequencies at which DPOAE levels decreased significantly were overtones of the stimulus frequency, suggesting a possible effect of acoustic stimulation. VEMP testing can be an invasive test method and should be performed with detailed consideration of the risks and benefits. The age factor can influence the decrease in DPOAE levels in VEMP testing.

## 1. Introduction

Vestibular evoked myogenic potential (VEMP) testing is a common clinical testing method used to evaluate the function of the saccule and the utricle [1]. VEMP testing, along with caloric testing and vHIT, is used to evaluate vestibular balance disorders [2]. VEMP testing is valuable for evaluating both central and peripheral vestibular disorders and is particularly important in diagnosing inferior vestibular nerve neuritis [3].

There are several vestibular balance disorders that have been studied for the utility of VEMP testing, including superior semicircular canal dehiscence (SSCD) [4], vestibular migraine (VM) [5,6], acoustic neuroma (vestibular schwannoma) [7], bilateral vestibulopathy (BVP) [8], and Ménière’s disease (MD) [9,10]. VEMP testing, especially cVEMP, is sensitive in detecting SSCD, because the third window effect results in an abnormally low VEMP threshold [11]. Studies suggest that VEMP abnormalities may be present in patients with VM, which may indicate possible brainstem involvement and help differentiate VM from MD [12,13]. VEMP testing can reveal decreased or absent responses in BVP patients and can aid diagnosis when other vestibular function tests are inconclusive [14].

The absence of asymmetry between the labyrinths, especially in the context of caloric testing, can lead to inconclusive results regarding bilateral lesions. VEMPs can help assess vestibular nerve function and detect subclinical vestibular involvement in patients with acoustic neuroma, even in the early stages [15]. However, VEMP testing uses strong sounds of around 120 to 130 dB SPL for the acoustic stimulus and considerations of the safe level of sound pressure that does not cause sensorineural hearing loss are still being studied [16]. One case of bilateral sensorineural hearing loss after VEMP testing has been previously reported in the literature [17], and a case of permanent bilateral sensorineural hearing loss after VEMP testing is also reported [18]. VEMP testing utilizes high-intensity stimuli, which may pose a risk of sensorineural hearing loss by damaging the outer hair cells (OHCs). Distortion product otoacoustic emission (DPOAE) is a sound generated by the cochlea in response to two simultaneous pure-tone stimuli, reflecting OHC function [19]. They serve as a non-invasive measure of cochlear (specifically OHC) function and are valuable in both diagnostic and screening audiology [20].

The previous studies examining the safe levels of sound pressure in VEMP testing using DPOAE focused on younger subjects (the age range of 20 to 35 years [21], 18 to 50 years [22], 25 to 42 years [23], and 18 to 35 years [24]), whereas VEMP testing in vestibular examinations is often performed on elderly subjects, and there are very few studies of the changes in the DPOAE level after VEMP testing in clinical vestibular function examinations, which often involve elderly patients attending outpatient clinics. As individuals age, high-frequency hearing loss commonly occurs, often due to damage to the OHCs in the inner ear [25]. Additionally, certain vestibular disorders, such as Ménière’s disease, can contribute to sensorineural hearing loss.

The purpose of this study is to assess the effect of sound stimulations used in VEMP testing during vestibular examinations on the reduction in DPOAE levels in a vertigo outpatient clinic.

## 2. Methods

This is a retrospective study conducted in a single institution. Patients referred to the vertigo outpatient clinic, Department of Otolaryngology at the JR Tokyo General Hospital between June 2021 and December 2024 were included in this study. This study protocol was reviewed and approved by the Research Ethics Committee of JR Tokyo General Hospital (approval number: R06-21, approval date: 27 November 2024). The information for this study was disclosed, and the participants could choose to opt out. An opt-out informed consent protocol was used for the use of participant data for research purposes. This consent procedure was reviewed and approved by the Research Ethics Committee of JR Tokyo General Hospital.

### 2.1. VEMP

The cervical vestibular evoked myogenic potential (cVEMP) and ocular VEMP (oVEMP) were recorded with the Nicolet EDX EMG/NCS/EP/IOM system (Natus Medical Inc., Middleton, WI, USA) using a tone burst stimulus of 500 Hz (rise, 1 ms; plateau, 2 ms; and fall, 1 ms) and a tone burst of 1 kHz (rise, 1 ms; plateau, 2 ms; and fall, 1 ms). The total stimulation count in a series of VEMP tests was limited to a maximum of 240 counts for each ear to comply with L_Aeq,8h_ 85 dB SPL. The stimulation sound pressure levels were 133 [26], 130 [14], 128 [27], or 125 dBpSPL [24] depending on the examination date, because a case of sudden bilateral sensorineural hearing loss after VEMP testing was reported [18] and the stimulation levels were gradually decreased after the reconsideration of the safe levels. The sound pressure levels were calibrated by the manufacturer.

In the cVEMP testing, electromyographic (EMG) activity was recorded from a surface electrode placed on the upper half of each sternocleidomastoid muscle (SCM), with a reference electrode on the side of the upper sternum and a ground electrode on the chin. During the recording, in the supine position, subjects were instructed to raise their heads from the pillow in order to contract the SCM. The EMG signal from the stimulated side was amplified and bandpass-filtered (20–2000 Hz). The stimulation rate was 5.1 Hz, and the analysis time was 100 ms. The responses to 20 stimuli were averaged from three times [28].

In the oVEMP testing, the subjects laid supine on a bed, with their head supported by a pillow and with surface EMG electrodes placed on the skin 1 cm below (active) and 3 cm below (indifferent) the center of each lower eyelid. The ground electrode was placed on the chin. During testing, the subject looked up approximately 30 degrees straight ahead and maintained their focus on a small dot approximately 1 m from their eyes. The signals were amplified by a differential amplifier (bandwidth: 0.5–500 Hz). The responses to 20 stimuli were averaged from three times [28].

### 2.2. DPOAE

The DPOAE levels were measured from left to right before and within 1 min after VEMP testing using ILO V6 (Otodynamics Ltd., Hatfield, UK), with the conditions of 2f1-f2, f2/f1 ratio = 1.22, L1/L2 = 65/55 dB SPL, and 2 points per octave with 1/2 octave range in the range from 1 kHz to 6 kHz (1 kHz, 1.4 kHz, 2 kHz, 2.8 kHz, 4 kHz, and 6 kHz). The sum all 1/2 octave (Sum) is an overall DPOAE level added in the range from 1 kHz to 6 kHz above. The average 1/2 octave (1–6 kHz) (Average) is a mean DPOAE level in the range from 1 kHz to 6 kHz above. The DPOAE levels were measured routinely to evaluate vestibular balance disorders with sensorineural hearing loss and to monitor changes in cochlear function before and after VEMP. The order of VEMP testing was cVEMP—left, cVEMP—right, oVEMP—left, and oVEMP—right. The decreased DPOAE level is calculated with post–pre VEMP. The negative values indicate deterioration, whereas the positive values indicate improvement.

There were few cases without data of the sum all 1/2 octave and average 1/2 octave (1–6 kHz) because of the calculation algorithm. An otoscopy was performed prior to the examination to rule out abnormalities in the outer and middle ears.

### 2.3. Statistical Analysis

Excel for Microsoft 365 (version 2207, Microsoft, Redmond, WA, USA) was used for processing the data. The paired *t*-test, Wilcoxon signed-rank test, Pearson product moment correlation coefficient analysis, *t*-test, and analysis of variance (ANOVA) were applied after confirming the Kolmogorov–Smirnov normality test (*p* > 0.05) and equality of variances using the Levene’s test (*p* > 0.05) using R version 4.4.2 software (R Core Team; R Foundation for Statistical Computing, Vienna, Austria, 2024) with devtools, ggplot2, Rmisc, readxl, exactRankTests, and rstatix packages. *p*-values less than 0.05 were considered statistically significant.

The DPOAE levels are presented as the mean ± standard deviation [dB SPL] in all the figures and tables. The box plot illustrates the distribution of the data through several key statistics: the median (indicated by the line within the box), the first quartile (Q1, the lower edge of the box), and the third quartile (Q3, the upper edge of the box). The “whiskers” extend to the minimum and maximum values within 1.5 times the interquartile range (IQR) from the quartiles. Data points outside this range are considered outliers and are displayed as individual points beyond the whiskers.

## 3. Results

A total of 174 patients (80 males and 94 females; median age, 53 years [interquartile range (IQR), 39–67 years; range, 15–85 years]) were examined in the vertigo outpatient clinic and there were no cases in which the DPOAE test could not be performed. All cases referred to the vertigo outpatient clinic were tested for both cVEMP testing and oVEMP testing with air conduction stimulation at 500 Hz and 1 kHz. There were no cases with conductive hearing loss, including the perforation of the tympanic membrane. The case characteristics and diagnosis are shown in Table A1.

**Figure A1:**
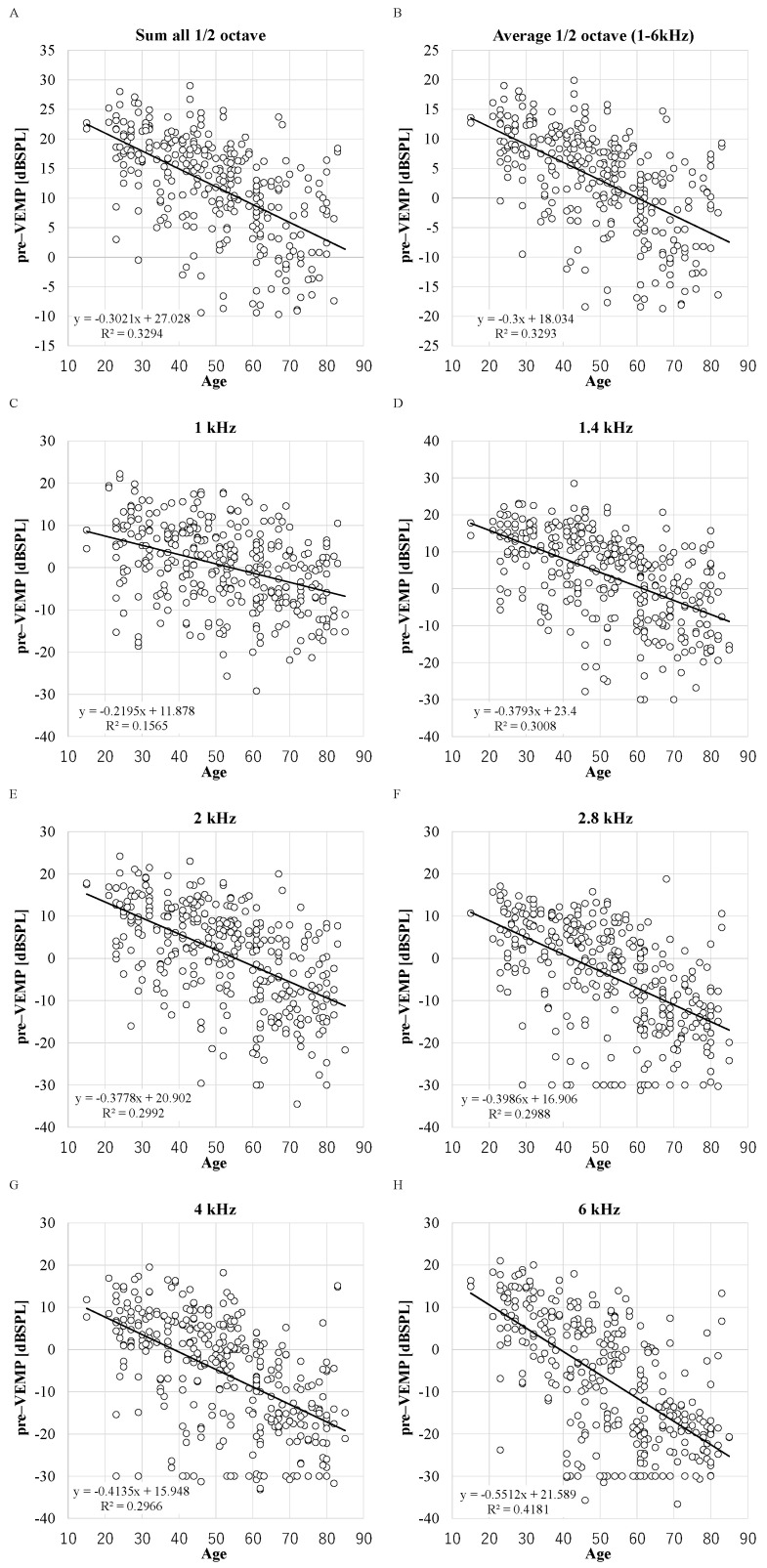
The distribution between DPOAE levels before VEMP testing and age. X-axis: ages in years. R^2^: the coefficient of determination. *p*-values of Pearson product moment correlation coefficient analysis: *p* < 0.0001 in all parameters. (*n* = 348 ears in 1, 1.4, 2, 2.8, 4, and 6 kHz and *n* = 298 in sum and average). (**A**): sum; (**B**): average; (**C**): 1 kHz; (**D**): 1.4 kHz; (**E**): 2 kHz; (**F**): 2.8 kHz; (**G**): 4 kHz; (**H**): 6 kHz.

The DPOAE levels before VEMP testing showed significant differences between males and females and between the older group aged 65 years or over and the younger group (*p* < 0.05, *t*-test) (*p* < 0.05, ANOVA) (Table A2). Females and the younger group presented higher levels. The negative correlations between the DPOAE levels before VEMP testing and age were also significant in all frequencies (*p* < 0.0001) (Figure A1). The stimulation levels for VEMPs were 133, 130, 128, or 125 dBpSPL and the number of cases at each stimulation level was 15, 18, 15, and 126, respectively (Table A3).

**Figure A2:**
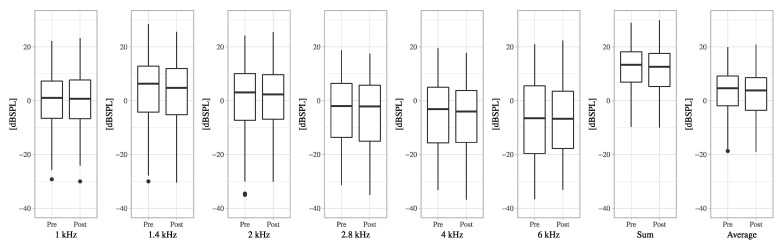
The boxplots of DPOAE levels of pre-VEMP and post-VEMP for each frequency.

**Figure A3:**
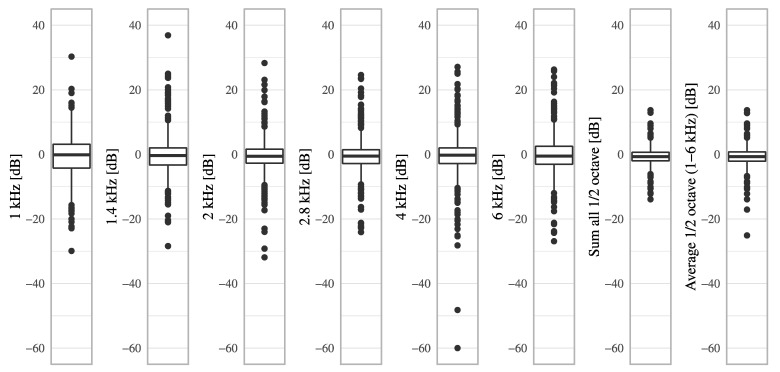
The boxplots of DPOAE level decreases from pre-VEMP to post-VEMP for each frequency.

The DPOAE levels decreased significantly after VEMP testing at 1.4 kHz, 2 kHz, 2.8 kHz, sum all 1/2 octave, and average 1/2 octave (1–6 kHz) (Table 1, Figure A2 and Figure A3). The decreases in the average DPOAE level at the average 1/2 octave (1–6 kHz) were significant in 125 dBpSPL and 130 dBpSPL stimulations in a subgroup analysis (*p* < 0.05) (Table A3). The decreases in the DPOAE level at the average 1/2 octave (1–6 kHz) did not significantly differ between each stimulation level, but the decrease tended to be larger at higher stimulation levels (−0.9, −0.8, −0.8, or −0.7 dB in 133, 130, 128, or 125 dBpSPL, respectively) (*p* < 0.05) (Table A3).

**Figure 1:**
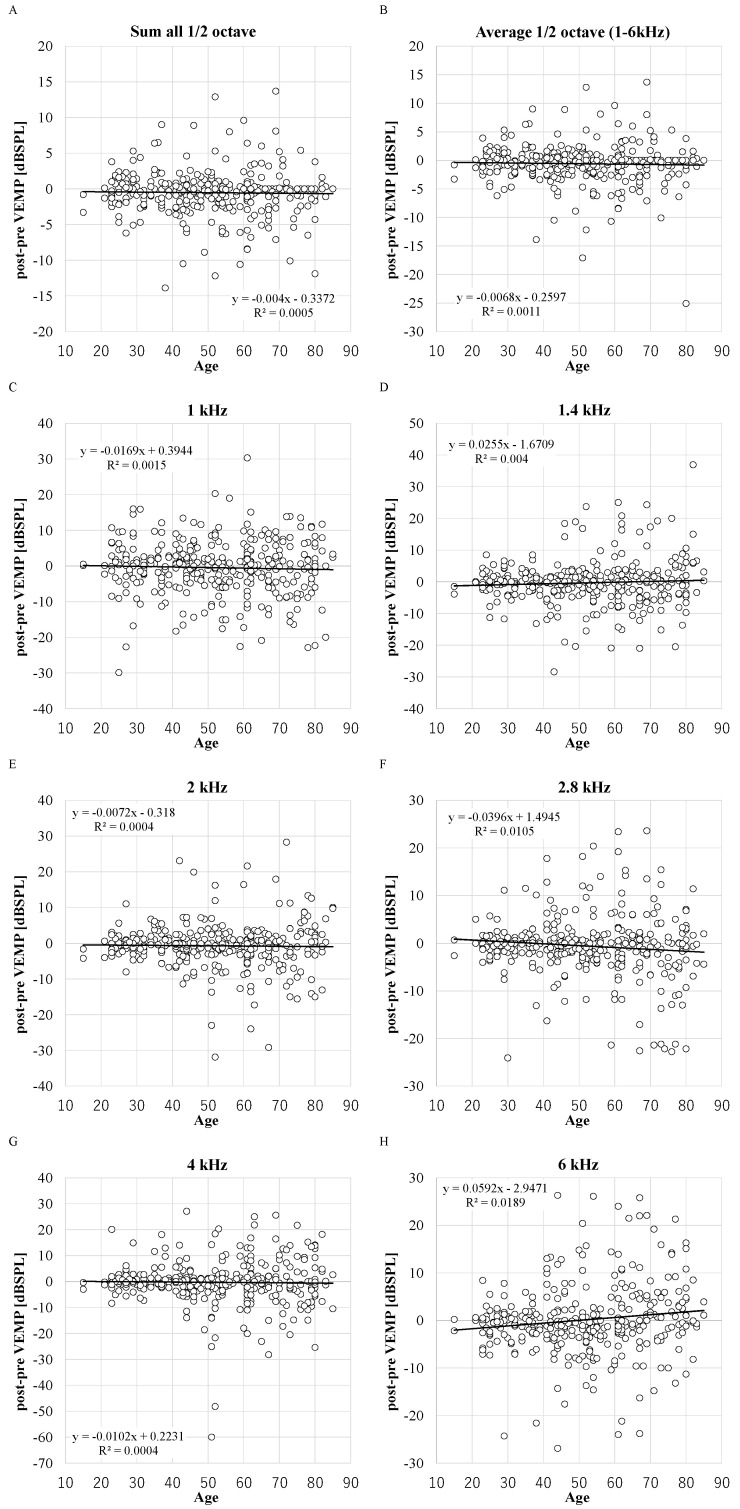
The distribution of decreased DPOAE levels and age. X-axis: ages in years. R^2^: the coefficient of determination. *p*-values of Pearson product moment correlation coefficient analysis: *p* = 0.45, *p* = 0.83, *p* = 0.95, *p* = 0.06, *p* = 0.44, *p* = 0.01, *p* = 0.15, and *p* = 0.20 in 1, 1.4, 2, 2.8, 4, and 6 kHz, sum and average. (*n* = 348 ears in 1, 1.4, 2, 2.8, 4, and 6 kHz and *n* = 298 in sum and average). (**A**): sum; (**B**): average; (**C**): 1 kHz; (**D**): 1.4 kHz; (**E**): 2 kHz; (**F**): 2.8 kHz; (**G**): 4 kHz; (**H**): 6 kHz.

**Figure A4:**
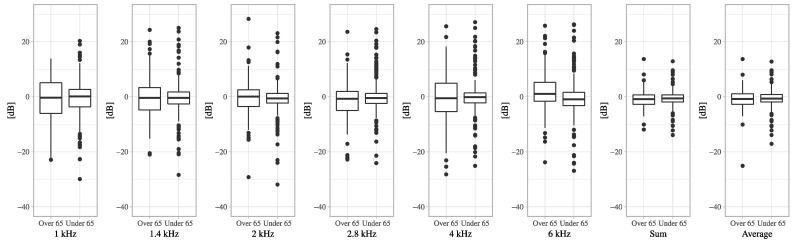
The boxplots of DPOAE level decreases from pre-VEMP to post-VEMP in the older group aged 65 years or over and the younger group for each frequency.

The positive correlation between age and the increased DPOAE levels at 6 kHz was significant (DPOAE level = 0.0592 × Age − 2.9471, R^2^ = 0.0189, *p* = 0.01) (Figure 1). The significant increase in the DPOAE levels at 6 kHz in the older group aged 65 or over was also observed (*p* = 0.024) (Table 2). The significant decreases in the DPOAE levels were observed at 2.8 kHz in the older group, and at 1.4 kHz, 2 kHz, 2.8 kHz, 6 kHz, sum all 1/2 octave, and average 1/2 octave (1–6 kHz) in the younger group (Table 2, Figure A4). The decrease in the average 1/2 octave (1–6 kHz) was larger in the older group (−0.9 or −0.7 dB in the older or younger group, respectively), whereas there was no significant difference between the older group and the younger group (Table 2, Figure A4).

**Figure 2:**
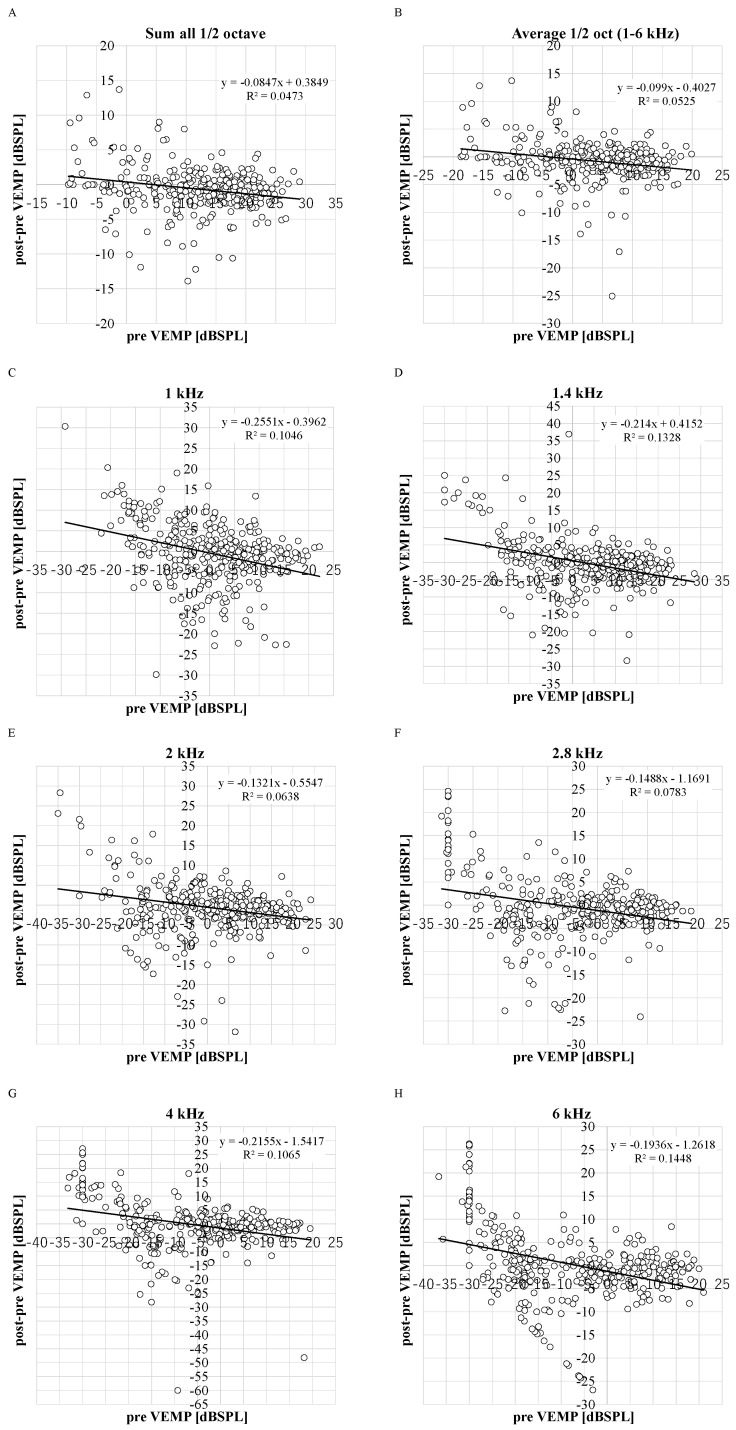
The distribution between decreased DPOAE levels and DPOAE levels before VEMP testing. R^2^: the coefficient of determination. *p*-values of Pearson product moment correlation coefficient analysis: *p* < 0.0001, *p* < 0.0001, *p* = 0.0015, *p* < 0.0001, *p* < 0.0001, *p* < 0.0001, *p* = 0.03, and *p* = 0.01 in 1, 1.4, 2, 2.8, 4, and 6 kHz, sum and average. (*n* = 348 ears in 1, 1.4, 2, 2.8, 4, and 6 kHz and *n* = 298 in sum and average). (**A**): sum; (**B**): average; (**C**): 1 kHz; (**D**): 1.4 kHz; (**E**): 2 kHz; (**F**): 2.8 kHz; (**G**): 4 kHz; (**H**): 6 kHz.

**Figure A5:**
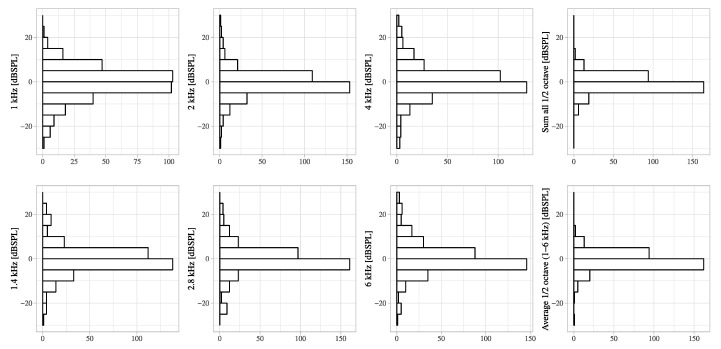
The distribution of DPOAE level decreases from pre-VEMP to post-VEMP for each frequency. The X-axis shows the number of cases.

The negative correlations between the DPOAE levels before VEMP testing and the decrease in the DPOAE level were significant in all frequencies (R^2^ = 0.1946, 0.1328, 0.0638, 0.0783, 0.1065, 0.1448, 0.0473, and 0.0525 in 1, 1.4, 2, 2.8, 4, and 6 kHz, sum and average) (*p* < 0.05) (Figure 2). The average DPOAE levels at the average 1/2 octave (1–6 kHz) decreased by more than 5 dB in 9% of ears (Figure A5), and the average DPOAE levels before VEMP testing in these ears (3.0 ± 7.3 dB SPL) was not significantly different from the other ears (3.6 ± 8.2 dB SPL). Although there is no standard for significant changes in DPOAE changes, because 5 dB steps are used in audiometric measurements, and because there is a criterion that if the difference between threshold values measured with and without masking in audiometric tests using masking is within 5 dB, the value without masking is adopted, the 5 dB difference is treated as an important indicator in measurement procedures and evaluation criteria, and thus 5 dB was appropriated as an obvious change.

The DPOAE levels at 2.8 kHz, sum all 1/2 octave, and average 1/2 octave (1–6 kHz) showed a significant decrease for both males and females in a subgroup analysis (male: *p* = 0.032, female: *p* = 0.018; male: *p* = 0.006, female: *p* < 0.001; and male: *p* = 0.010, female: *p* < 0.001) (Table A4). In addition, the DPOAE levels at 2 kHz decreased significantly only in females. There were significant decreases in DPOAE levels at the sum all 1/2 octave and average 1/2 octave (1–6 kHz) in the left ear, and at 2 kHz, 2.8 kHz, sum all 1/2 octave, and average 1/2 octave (1–6 kHz) in the right ear in a subgroup analysis (Table A5). Only the age factor significantly affected the changes in DPOAE levels at 2.8 kHz and 6 kHz in the ANOVA (Table 3).

## 4. Discussion

The DPOAE levels decreased significantly after VEMP testing in this study, as has been previously reported, suggesting that the acoustic stimulation of the VEMP test can induce inner ear damage. The frequencies at which DPOAE levels decreased significantly were overtones of the stimulus frequency, suggesting a possible effect of acoustic stimulation.

Sound stimulation with 125 dB pSPL caused a significant reduction in the DPOAE levels in this study. The recommended maximum sound pressure for VEMP testing was 140 dB pSPL in the initial international guideline, including attention to L_Aeq,8h_ [29]; however, the recommended maximum safety level was reduced to 126 dB pSPL later [16]. The sound stimulation can cause a decrease in DPOAE levels even if the sound pressure is below the current maximum indication in the guidelines.

Krause et al. reported that DPOAE levels in 30 subjects aged between 20 and 35 years exposed to VEMP sound stimulation (133 dB SPL) had decreased significantly 5 min after VEMP testing. A decrease observed after a 5 min interval indicates more temporary changes. The decrease ranged from 0.5 to 3.0 dB in the high-frequency range between 4 kHz and 8 kHz and recovered after 24 h [21]. Colebatch et al. posted a letter in response to this article discussing the conditions used in their VEMP test, in which the duration of the stimulation was relatively longer (10 ms) than commonly used and the L_Aeq,8h_ was over the safety level, and concluded that the sound stimulations in VEMP testing for diagnostic purposes are unlikely to cause damage to the cochlea provided that peak intensity limits are not exceeded [30]. However, Krause et al. responded that a Hann window was applied to reduce the noise impact and that researchers and doctors should recognize the potential risk of adverse effects in the cochlea due to VEMP testing [30]. Strömberg et al. reported a significant decrease in DPOAE levels ranging from 0.5 to 1.35 dB at 0.75 kHz and from 1.6 to 2.1 dB at 3 kHz after VEMP testing with 130 dB peSPL in 24 patients aged between 18 and 50 years with a mean age of 37 years [22]. Rodriguez et al. studied 15 children aged between 4 and 12 years and 10 adults aged between 21 and 37 years who underwent VEMP testing with 125 dB SPL in adults and 120 dB SPL in children and found no significant decrease in DPOAE levels, and they recommended 120 dB SPL stimulation [23], which is below the normal threshold in the elderly [31,32]. Singh et al. studied 60 adults in the age range of 18 to 35 years divided into a control group and a VEMP-tested group and found no significant changes in OAE levels after VEMP testing with 125 dB peSPL stimulation compared to the control group [24]. These studies focused on younger subjects, whereas vestibular examinations are often performed on older people. Elderly people may be more vulnerable to loud sounds due to a variety of factors, and the safety level they require may be lower than that in younger people [33,34,35]. Elderly people may already have high-frequency sensorineural hearing loss due to aging. These studies show that the acoustic stimulation used in VEMP testing can temporarily affect DPOAE levels in young subjects depending on the stimulation sound level, although the effect in the elderly is still unknown. In this study, the significant negative correlation between age and the decrease in OAE levels was observed at 2.8 kHz in ANOVA. The aging factor may contribute to exacerbating the decline in DPOAE levels. Several previous reports with young subjects have reported no significant decrease in the OAE level, so an aging factor can also contribute to the decrease in the OAE level after VEMP testing. However, the significant positive correlation between the decrease in DPOAE level at 6 kHz and age was observed, which may be related to the negative correlation between age and the DPOAE levels before VEMP testing because there was also the negative correlation between the DPOAE levels before VEMP testing and the decrease in DPOAE levels. The sound pressure that does not cause a decrease in DPOAE levels is expected to be below 125 dB SPL; however, the normal threshold for cVEMP testing for subjects in their sixties is reported to be 110–125 dB SPL [31,32], so it is likely that VEMP testing cannot be properly performed from a strict safety standpoint. The decrease in DPOAE level tended to be smaller with lower stimulation levels, but these differences were not significant, and a prediction of the safe level based on these values seems difficult. There is no evidence that the elderly are more vulnerable to noise [36]. Comparisons by age show rather an inverted U-shaped relationship, indicating that as far as annoyance and sleep disturbance are concerned, both the young and the elderly are at lower risk; however, perhaps the elderly are more vulnerable with respect to cardiovascular effects, which may be a combined effect of air pollution and noise [37]. Further investigation into the methodology of VEMP is beneficial to determine the optimal lowest stimulus intensity.

In this study, the decreases in the DPOAE level at 2 kHz and 2.8 kHz were significant only in the right ear in a subgroup analysis. Asymmetrical hearing loss with left-sided hearing loss in noise exposure is reported [38,39,40], and physiological aspects or a head shadowing effect have been suggested but are yet to be proven [41]. Because the VEMP testing was performed from left to right and DPOAE were recorded soon after VEMP testing in this study, the elapsed time from VEMP testing to DPOAE testing may have affected the decrease in DPOAE levels. The interval between VEMP testings of the left and right ear and the interval between DPOAE testings of the left and right ear are generally comparable and may not have had a significant effect. The results of this study showed that DPOAE levels worsened less in the ear in which VEMP was first tested (left ear), which suggests that DPOAE may recover in a short time and that the deterioration in DPOAE levels may be a transient phenomenon.

The recovery functions for noise exposure elapsed time and DPOAE changes were reported in several studies. In a study in which 39 normal-hearing human subjects were monaurally exposed to a 1 kHz tone lasting for 3 min at an equivalent threshold sound pressure level of 105.5 dB, the exponential regression equation fitted to the data is as follows: DPOAE shift (916–2380 Hz) [dB] y = 4.06 × e^−t/0.70^ + 3.14 × e^−t/10.24^ (t: [min]) [42]. In a study in which 16 normal-hearing human subjects were exposed to a moderate monaural sound exposure of 10 min to a 2 kHz tone normalized to an exposure level L_EX,8h_ of 80 dB(A), DPOAEs were measured before and in the following 70 min after the exposure in a 1/3-octave band centered at 3 kHz and the recovery functions were y = 1.63 × e^−t/3.33^ + 3.45 × e^−t/47.6^ (monaural pure tone, L_EX,8h_ = 80 dB(A)), y = 2.06 × e^−t/6.28^ + 1.93 × e^−t/56.8^ (continuous broad-band noise, L_EX,8h_ = 80 dB(A)), and y = 1.82 × e^−t/20^ (continuous broad-band noise + impulsive noise, L_peak_ = 117 dB(C)) [43]. If the time is 5 min, the results of each equation above are −4.2, −7.6, −4.9, and −1.2, respectively. In this study, there was no significant difference of decreased DOPAE levels between the left and right ear, but the mean difference was around 1 at 2 kHz, which is close to the value of the recovery function of continuous broad-band noise + impulsive noise [43]. In summary, studies on DPOAE recovery after noise exposure revealed exponential recovery patterns.

The DPOAE levels at 2 kHz showed a significant decrease only for females in a subgroup analysis in this study, whereas the higher risk of hearing loss in males than in females is reported in previous studies [38,44,45,46,47,48]. Although these lateral differences and sex differences were not significant in ANOVA, these results of subgroup analyses may reflect the fact that the sound stimulation used in VEMP is large enough to influence cochlear function. Males and females may have inherent structural or functional differences in the vestibular or cochlear systems, such as variations in otolith organ size, nerve conduction properties, or inner ear fluid dynamics [49]. Sex hormones, particularly estrogen and progesterone, are known to influence auditory function [50,51]. Hormonal fluctuations across the menstrual cycle or long-term hormonal differences between sexes could impact sensitivity to noise. Variations in skull thickness, middle ear resonance, or impedance between males and females could affect how sound stimuli reach the inner ear, potentially influencing sound stimuli [52]. The intense acoustic stimulation may have subtle differential effects on cochlear function between sexes, potentially due to differences in OHC function or susceptibility to sound-induced changes [53].

The frequencies at which significant decreases in DPOAE levels occurred were 1.4 kHz, 2 kHz, and 2.8 kHz, which were higher than the stimulated frequencies. In noise-induced hearing loss, the characteristic frequency of the most damaged region in the cochlea is approximately half an octave above the frequency of the acoustic stimulus in the animal and stimulation studies [54,55,56,57], and this “half-octave shift” phenomenon is also observed in the several clinical studies [58,59]. This phenomenon was firstly observed in a study of TTS in which human subjects were exposed to 110, 120, and 130 dB SPL of pure tones at 1000 and 2000 Hz for 1 to 64 min [60]. The threshold increases in the frequency range were limited to the exposed and higher frequencies, with a maximum loss of 50 to 60 dB occurring half an octave above the exposed tone. In a study in which 60 normal-hearing human subjects were exposed to an octave-band noise centered at 4, 2, 1, or 0.5 kHz for 16–24 h in a diffuse sound field, TTS increased for about 8 h and then reached a plateau or asymptote [61]. The relation between TTS and exposure duration can be described by a simple exponential function with a time constant of 2.1 h, and the threshold shifts were maximal about 1/2 an octave above the center frequency of the noise. This phenomenon was also observed in the DPOAE study, where the amplitude was reduced in a frequency-specific manner, with the greatest reduction occurring approximately half an octave above the noise frequency [62]. In a study evaluating both transient-evoked otoacoustic-emission (TEOAE) and behavioral hearing thresholds before and after the sound exposure of 105 dB SPL half-octave band of noise centered at 1.414 kHz for 10 min in 14 normal-hearing subjects, the maximum temporary emission shifts were half to 1 octave above the exposure frequency [63]. The results in this study are consistent with this phenomenon, but the characteristic frequency of the region in the cochlea most affected by the acoustic stimulation used in VEMP needs to be further examined in detail. This “half-octave shift” phenomenon may be involved in the function of OHC in the cochlea [64].

In the present study, pure tone audiometry (PTA) thresholds were not evaluated because OAE is more sensitive than PTA. In a study of 285 U.S. Marine Corps recruits measuring audiometric thresholds and OAE before and 3 weeks after exposure to impulse noise sources from weapons’ fire and simulated gunfire, and 32 non-exposed controls measuring audiometric thresholds and OAE, there was essentially no association between significant threshold shift (STS) and significant emission shift (SES), SES was more common than STS, and the SES ear group had more STS ears than the group without SES ears, indicating the high sensitivity of OAEs in comparison to audiometric thresholds and the association of low levels of OAEs with a future risk of hearing loss [65].

This study has some limitations. Firstly, this was a retrospective study conducted at a single institution. Second, the PTA testing including chronological changes to evaluate the associations between DPOAE levels and temporary and permanent threshold shift was not evaluated due to the time limitation of the examination. Third, there is no control group, and a crossover study or other evaluation method should be considered for further assessments. Forth, the number of cases is small, and further study is needed to confirm lateral differences and sex differences. The diverse range of vestibular balance disorders’ cases limited the interpretation of the results. The results of this study can be considered important in terms of actual clinical practice. Finally, the DPOAEs were only recorded just before VEMP testing and chronological changes were not evaluated. There may be potential long-term effects on hearing, which should be further explored in prospective studies. The VEMP may pose a risk only if it leads to lasting changes in hearing.

VEMP testing can cause a noise-induced transient threshold shift, as well as noise-induced permanent threshold shifts in rare cases with high sensitivity to noise exposure. The decreased DPOAE level after VEMP testing varied widely between cases, indicating that the tolerance to sound stimulation differs greatly from person to person, and VEMP testing would not be appropriate in a patient with low tolerance to sound stimulation. During the VEMP testing, the examiner should take care of the patient if they experience acoustic disturbances caused by sound stimulation, and the efficacy and safety of the VEMP test should be carefully considered before performing the examination. To prevent acoustic trauma, it is necessary to take measures such as exposing the subject to the stimulus sound before starting the test, allowing breaks during the test, and reducing the number of stimuli or the sound pressure level of the stimulus. ABR testing also uses strong sounds, which can also cause acoustic trauma, so the same cautions should be considered for ABR testing.

## 5. Conclusions

The strong sound stimulation used in VEMP testing can decrease DPOAE levels. VEMP testing can be an invasive test method and should be performed with detailed consideration of the risks and benefits. The age factor can influence the decrease in DPOAE levels in VEMP testing.

## Figures and Tables

**Table 1:** The average DPOAE levels before and after VEMP testing for each frequency. *p*-value: *p*-values of paired *t*-test (paired) and Wilcoxon signed-rank (Wilcoxon). Mean ± standard deviation. (*n* = 348 ears in 1, 1.4, 2, 2.8, 4, and 6 kHz and *n* = 298 in sum and average) *: *p* < 0.05.

	1 kHz	1.4 kHz	2 kHz	2.8 kHz	4 kHz	6 kHz	Sum All 1/2 oct	Average 1/2 oct (1–6 kHz)
pre–VEMP	0.4 ± 9.5	3.5 ± 11.9	1.1 ± 11.9	−4.0 ± 12.5	−5.7 ± 13.0	−7.3 ± 14.6	11.9 ± 8.6	3.0 ± 8.5
post–VEMP	−0.1 ± 10.0	3.2 ± 11.4	0.4 ± 11.9	−4.5 ± 12.4	−6.0 ± 13.1	−7.1 ± 13.7	11.0 ± 8.8	2.1 ± 8.7
post–pre	−0.5 ± 7.5	−0.3 ± 7.0	−0.7 ± 6.2	−0.6 ± 6.7	−0.3 ± 8.6	0.1 ± 7.4	−0.6 ± 3.4	−0.7 ± 3.7
*p*-value (paired)	0.111	0.181	0.018 *	0.053	0.25	0.644	<0.001 *	<0.001 *
*p*-value (Wilcoxon)	0.472	0.037 *	0.005 *	0.004 *	0.273	0.291	<0.001 *	<0.001 *

**Table 2:** The difference in DPOAE level for each frequency in older (≥65 years of age) and younger (<65 years of age) age groups. *p*-value: *p*-values of paired *t*-test (paired) and Wilcoxon signed-rank (Wilcoxon). Mean ± standard deviation. *: *p* < 0.05, n: number of ears.

	1 kHz	1.4 kHz	2 kHz	2.8 kHz	4 kHz	6 kHz	Sum All 1/2 oct	Average 1/2 oct (1–6 kHz)	n
≥65	−1.0 ± 8.2	0.1 ± 8.6	−0.5 ± 7.5	−2.1 ± 8.3	−0.4 ± 9.6	1.9 ± 8.3	−0.7 ± 4.1	−0.9 ± 5.0	96
*p*-value (paired)	0.114	0.53	0.241	0.008 *	0.33	0.988	0.097	0.092	
*p*-value (Wilcoxon)	0.466	0.491	0.432	0.029 *	0.574	0.024 *	0.056	0.071	
<65	−0.3 ± 7.2	−0.5 ± 6.2	−0.8 ± 5.6	0.0 ± 5.8	−0.3 ± 8.2	−0.5 ± 7.0	−0.6 ± 3.2	−0.7 ± 3.4	252
*p*-value (paired)	0.261	0.104	0.016 *	0.493	0.304	0.114	0.001 *	<0.001 *	
*p*-value (Wilcoxon)	0.653	0.041 *	0.003 *	0.037 *	0.344	0.003 *	<0.001 *	<0.001 *	
								Total	348

**Table 3:** The ANOVA of factors in the decreased DPOAE levels. L: left, R: right. ≥65/<65: older (≥65 years of age) and younger (<65 years of age) age groups. *: *p* < 0.05 (*n* = 348 ears in 1, 1.4, 2, 2.8, 4, and 6 kHz and *n* = 298 in sum and average).

	1 kHz	1.4 kHz	2 kHz	2.8 kHz	4 kHz	6 kHz	Sum All 1/2 oct	Average 1/2 oct (1–6 kHz)
L/R	F(1,343) = 0.071,	F(1,343) = 0.061,	F(1,343) = 1.924,	F(1,343) = 0.502,	F(1,343) = 2.478,	F(1,343) = 0.000,	F(1,293) = 0.015,	F(1,293) = 0.039,
	*p* = 0.790	*p* = 0.805	*p* = 0.166	*p* = 0.479	*p* = 0.116	*p* = 0.983	*p* = 0.902	*p* = 0.843
Male/Female	F(1,343) = 0.093,	F(1,343) = 0.350,	F(1,343) = 0.027,	F(1,343) = 0.542,	F(1,343) = 0.174,	F(1,343) = 1.330,	F(1,293) = 0.003,	F(1,293) = 0.179,
	*p* = 0.760	*p* = 0.554	*p* = 0.868	*p* = 0.462	*p* = 0.677	*p* = 0.250	*p* = 0.955	*p* = 0.672
dBpSPL	F(1,343) = 0.539,	F(1,343) = 0.007,	F(1,343) = 0.936,	F(1,343) = 2.372,	F(1,343) = 0.064,	F(1,343) = 0.798,	F(1,293) = 0.366,	F(1,293) = 0.175,
	*p* = 0.463	*p* = 0.933	*p* = 0.334	*p* = 0.124	*p* = 0.801	*p* = 0.372	*p* = 0.546	*p* = 0.676
≥65/<65	F(1,343) = 0.575,	F(1,343) = 0.444,	F(1,343) = 0.125,	F(1,343) = 6.347,	F(1,343) = 0.021,	F(1,343) = 8.171,	F(1,293) = 0.022,	F(1,293) = 0.138,
	*p* = 0.449	*p* = 0.505	*p* = 0.724	*p* = 0.012*	*p* = 0.884	*p* = 0.005 *	*p* = 0.881	*p* = 0.711

## Data Availability

The data that support the findings of this study are available from the corresponding author, upon reasonable request with the permission of the research ethics committee.

## Appendix A

**Table A1:** Case characteristics and diagnosis. Age: mean (standard deviation).

Characteristics			
Sex			n = 174
	Male	80	
	Female	94	
Age		52.4 (17.2)	n = 174
**Diagnosis**			
Functional (psychogenic) dizziness			48
Vestibular migraine			9
Migraine			7
Tension headache			1
Orthostatic dysregulation			11
Benign paroxysmal positional vertigo (BPPV)			39
Ménière’s disease			
	Definite		13
	Probable		15
Low-tone sensorineural hearing loss			1
Ramsay Hunt syndrome			3
Sudden unilateral hearing loss and vertigo			2
Vestibular neuritis			11
Peripheral vestibular dysfunction			7
Idiopathic bilateral vestibulopathy (IBV)			1
Sensory ataxia			1
Acoustic neuroma			1
Cerebellar disorders			2
Petroclival meningioma			1
Normal			1
		Total	174

**Table A2:** The DPOAE levels before VEMP testing. (**A**): The average DPOAE levels for each parameter and *p*-values of the *t*-test. Mean ± standard deviation. (**B**): The ANOVA of factors in the DPOAE levels before VEMP testing. ≥65/<65: older (≥65 years of age) and younger (<65 years of age) age groups. *: *p* < 0.05.

A								
	1 kHz	1.4 kHz	2 kHz	2.8 kHz	4 kHz	6 kHz	Sum All 1/2 oct	Average 1/2 oct (1–6 kHz)
L	0.7 ± 9.1	3.9 ± 11.6	1.4 ± 11.8	−3.6 ± 12.5	−4.9 ± 12.5	−7.2 ± 14.8	12.0 ± 8.5	3.1 ± 8.4
R	−0.2 ± 10.2	2.9 ± 12.2	0.5 ± 12.0	−4.6 ± 12.4	−7.1 ± 13.8	−7.5 ± 14.3	11.8 ± 8.8	3.0 ± 8.7
*p*-value	0.793	0.518	0.336	0.297	0.825	0.252	0.388	0.348
Male	0.1 ± 8.5	3.0 ± 12.4	0.5 ± 12.2	−5.5 ± 12.8	−7.9 ± 12.3	−10.4 ± 13.5	10.9 ± 8.8	2.1 ± 8.7
Female	0.7 ± 10.3	4.0 ± 11.4	1.6 ± 11.6	−2.7 ± 12.1	−3.8 ± 13.3	−4.6 ± 15.0	12.8 ± 8.3	3.8 ± 8.3
*p*-value	0.272	0.217	0.193	0.021 *	0.001 *	<0.001 *	0.034 *	0.043 *
≥65	−4.3 ± 8.2	−4.9 ± 10.4	−7.4 ± 10.2	−12.9 ± 9.3	−15.0 ± 9.6	−18.2 ± 9.4	4.7 ± 8.2	−4.1 ± 8.3
<65	2.2 ± 9.4	6.8 ± 10.7	4.3 ± 10.8	−0.6 ± 11.9	−2.2 ± 12.4	−3.1 ± 14.1	13.9 ± 7.6	5.0 ± 7.5
*p*-value	<0.001 *	<0.001 *	<0.001 *	<0.001 *	<0.001 *	<0.001 *	<0.001 *	<0.001 *
**B**								
	**1 kHz**	**1.4 kHz**	**2 kHz**	**2.8 kHz**	**4 kHz**	**6 kHz**	**Sum All** **1/2 oct**	**Average 1/2** **oct (1–6 kHz)**
L/R	F(1,344) = 0.733,	F(1,344) = 0.003,	F(1,344) = 0.223,	F(1,344) = 0.356,	F(1,344) = 1.114,	F(1,344) = 0.597,	F(1,304) = 0.101,	F(1,304) = 0.189,
	*p* = 0.393	*p* = 0.960	*p* = 0.637	*p* = 0.551	*p* = 0.292	*p* = 0.440	*p* = 0.750	*p* = 0.664
Male/Female	F(1,344) = 0.394,	F(1,344) = 0.766,	F(1,344) = 0.940,	F(1,344) = 5.240,	F(1,344) = 11.125,	F(1,344) = 17.675,	F(1,304) = 4.194,	F(1,304) = 3.708,
	*p* = 0.531	*p* = 0.382	*p* = 0.333	*p* = 0.023 *	*p* < 0.001 *	*p* < 0.001 *	*p* = 0.041 *	*p* = 0.055
≥65/<65	F(1,344) = 35.279,	F(1,344) = 82.998,	F(1,344) = 83.891,	F(1,344) = 85.030,	F(1,344) = 84.843,	F(1,344) = 98.885,	F(1,304) = 72.487,	F(1,304) = 71.287,
	*p* < 0.001 *	*p* < 0.001 *	*p* < 0.001 *	*p* < 0.001 *	*p* < 0.001 *	*p* < 0.001 *	*p* < 0.001 *	*p* < 0.001 *

**Table A3:** The decreased DPOAE level for each frequency at each stimulation level. Mean ± standard deviation. n: number of ears, *: *p* < 0.05.

	1 kHz	1.4 kHz	2 kHz	2.8 kHz	4 kHz	6 kHz	Sum All 1/2 oct	Average 1/2 oct (1–6 kHz)	n
125dBpSPL	−0.3 ± 7.8	−0.3 ± 7.3	−0.5 ± 6.7	−0.2 ± 6.5	−0.3 ± 8.5	0.4 ± 7.5	−0.6 ± 3.5	−0.7 ± 3.9	252
*p*-value (paired)	0.28	0.225	0.145	0.282	0.302	0.822	0.008 *	0.006 *	
*p*-value (Wilcoxon)	0.839	0.048 *	0.083	0.093	0.321	0.702	<0.001 *	0.002 *	
128dBpSPL	−1.1 ± 7.0	−1.2 ± 6.1	−1.5 ± 4.4	−2.3 ± 4.7	−0.9 ± 6.8	−1.1 ± 6.0	−0.8 ± 3.1	−0.8 ± 3.1	30
*p*-value (paired)	0.21	0.143	0.036 *	0.008 *	0.234	0.157	0.12	0.12	
*p*-value (Wilcoxon)	0.247	0.219	0.065	0.007 *	0.556	0.062	0.055	0.056	
130dBpSPL	−1.0 ± 6.4	0.9 ± 5.3	−1.5 ± 4.9	−0.3 ± 8.7	1.1 ± 9.5	−0.2 ± 5.5	−0.8 ± 2.8	−0.8 ± 2.8	36
*p*-value (paired)	0.185	0.851	0.038 *	0.427	0.743	0.421	0.062	0.047 *	
*p*-value (Wilcoxon)	0.318	0.707	0.188	0.593	0.913	0.422	0.079	0.039 *	
133dBpSPL	−1.1 ± 6.2	−0.9 ± 6.3	−1.0 ± 3.6	−2.1 ± 6.6	−1.6 ± 9.6	−0.6 ± 9.8	−1.0 ± 3.7	−0.9 ± 3.7	30
*p*-value (paired)	0.179	0.218	0.073	0.047 *	0.188	0.374	0.114	0.118	
*p*-value (Wilcoxon)	0.296	0.635	0.223	0.092	0.831	0.988	0.2	0.238	
								Total	348

**Table A4:** The decreased DPOAE level for each frequency by sex. Mean ± standard deviation. n: number of ears, *: *p* < 0.05.

	1 kHz	1.4 kHz	2 kHz	2.8 kHz	4 kHz	6 kHz	Sum All 1/2 oct	Average 1/2 oct (1–6 kHz)	n
Male	−0.4 ± 7.0	−0.3 ± 6.9	−0.6 ± 6.3	−0.9 ± 6.9	−0.6 ± 9.6	0.5 ± 7.0	−0.8 ± 3.2	−1.1 ± 4.1	160
*p*-value (paired)	0.258	0.428	0.101	0.055	0.446	0.871	0.010 *	0.009 *	
*p*-value (Wilcoxon)	0.948	0.301	0.108	0.032 *	0.845	0.952	0.006 *	0.010 *	
Female	−0.6 ± 7.9	−0.6 ± 7.1	−0.8 ± 5.9	−0.6 ± 6.6	−0.6 ± 8.2	−0.4 ± 7.8	−0.6 ± 3.3	−0.7 ± 3.3	188
*p*-value (paired)	0.148	0.142	0.046 *	0.241	0.196	0.313	0.012 *	0.011 *	
*p*-value (Wilcoxon)	0.338	0.062	0.018 *	0.047 *	0.182	0.174	<0.001 *	<0.001 *	
								Total	348

**Table A5:** The decreased DPOAE levels for each frequency in the left or right ears. Mean ± standard deviation. L: left, R: right. *: *p* < 0.05 (*n* = 348 ears in 1, 1.4, 2, 2.8, 4, and 6 kHz and *n* = 298 in sum and average).

	1 kHz	1.4 kHz	2 kHz	2.8 kHz	4 kHz	6 kHz	Sum All 1/2 oct	Average 1/2 oct (1–6 kHz)
L	−0.4 ± 6.7	−0.4 ± 7.2	−0.2 ± 6.0	−0.3 ± 6.8	−1.0 ± 9.3	0.2 ± 7.3	−0.7 ± 3.3	−0.8 ± 3.7
*p*-value (paired)	0.226	0.213	0.301	0.263	0.071	0.611	0.007 *	0.007 *
*p*-value (Wilcoxon)	0.629	0.177	0.488	0.093	0.248	0.704	0.021 *	0.021 *
R	−0.6 ± 8.2	−0.2 ± 6.8	−1.2 ± 6.3	−0.8 ± 6.5	0.4 ± 7.8	0.1 ± 7.6	−0.6 ± 3.5	−0.7 ± 3.8
*p*-value (paired)	0.168	0.315	0.008 *	0.048 *	0.758	0.595	0.016 *	0.014 *
*p*-value (Wilcoxon)	0.573	0.106	0.002 *	0.019 *	0.667	0.263	<0.001 *	<0.001 *
